# Improvement in Lung Cancer Survival: 6-Year Trends of Overall Survival at Hungarian Patients Diagnosed in 2011–2016

**DOI:** 10.3389/pore.2021.603937

**Published:** 2021-04-30

**Authors:** Krisztina Bogos, Zoltan Kiss, Lilla Tamási, Gyula Ostoros, Veronika Müller, László Urbán, Nóra Bittner, Veronika Sárosi, Aladár Vastag, Zoltán Polányi, Zsófia Nagy-Erdei, Andrea Daniel, Zoltán Vokó, Balázs Nagy, Krisztián Horváth, György Rokszin, Zsolt Abonyi-Tóth, Zsófia Barcza, Gabriella Gálffy, Judit Moldvay

**Affiliations:** ^1^ National Korányi Institute of Pulmonology, Budapest, Hungary; ^2^ MSD Pharma Hungary Ltd, Budapest, Hungary; ^3^ Department of Pulmonology, Semmelweis University, Budapest, Hungary; ^4^ Matrahaza Healthcare Center and University Teaching Hospital, Matrahaza, Hungary; ^5^ Pulmonology Clinic University of Debrecen, Debrecen, Hungary; ^6^ Faculty of Medicine, University of Pécs, Pécs, Hungary; ^7^ Eötvös Loránd University, Budapest, Hungary; ^8^ RxTarget Ltd. Szolnok, Budapest, Hungary; ^9^ University of Veterinary Medicine Budapest, Budapest, Hungary; ^10^ Syntesia Ltd, Budapest, Hungary; ^11^ Pulmonology Hospital Törökbálint, Budapest, Hungary; ^12^ Ist Department of Pulmonology, National Korányi Institute of Pulmonology, Semmelweis University, Budapest, Hungary; ^13^ 2nd Department of Pathology, MTA-SE NAP, Brain Metastasis Research Group, Hungarian Academy of Sciences, Semmelweis University, Budapest, Hungary

**Keywords:** lung cancer, long-term survival, mortality, Hungary, survival

## Abstract

**Objective:** Lung cancer is one of the most common cancers worldwide and its survival is still poor. The objective of our study was to estimate long-term survival of Hungarian lung cancer patients at first time based on a nationwide review of the National Health Insurance Fund database.

**Methods:** Our retrospective, longitudinal study included patients aged ≥20 years who were diagnosed with lung cancer (ICD-10 C34) between January 1, 2011 and December 31, 2016. Survival rates were evaluated by year of diagnosis, patient gender and age, and morphology of lung cancer.

**Results:** 41,854 newly diagnosed lung cancer patients were recorded. Mean age at diagnosis varied between 64.7 and 65.9 years during study period. One- and 5-year overall survival rates for the total population were 42.2 and 17.9%, respectively. Survival was statistically associated with gender, age and type of lung cancer. Female patients (*n* = 16,362) had 23% better survival (HR: 0.77, 95% confidence interval (CI): 0.75–0.79; *p* < 0.001) than males (*n* = 25,492). The highest survival rates were found in the 20–49 age cohort (5Y = 31.3%) and if the cancer type was adenocarcinoma (5Y = 20.5%). We measured 5.3% improvement (9.2% adjusted) in lung cancer survival comparing the period 2015–2016 to 2011–2012 (HR: 0.95 95% CI: 0.92–0.97; *p* = 0.003), the highest at females <60 year (0.86 (adjusted HR was 0.79), interaction analysis was significant for age and histology types.

**Conclusion:** Our study provided long-term Lung cancer survival data in Hungary for the first time. We found a 5.3% improvement in 5-year survival in 4 years. Women and young patients had better survival. Survival rates were comparable to–and at the higher end of–rates registered in other East-Central European countries (7.7%–15.7%).

## Introduction

Lung cancer (LC) is a rapidly progressing, life-threatening disease [1], which constitutes a major disease burden [2, 3]. Despite continuous advances in surgical, radio- and systemic treatment procedures (like targeted and immunotherapy) over the past years, lung cancer still has one of the lowest survival rates among carcinomas, making it one of the most important health problems of the 21st century [4, 5].

Variations in cancer survival between countries of comparable affluence and health systems have prompted international efforts to better understand the root of these differences. One–if not the main–reason for poor lung cancer survival may be late diagnosis [6]. Overall, the 5-year lung cancer survival rate is one of the poorest among cancers. The 5-year survival of LC, at all stages combined, varied between 10 and 20% in Europe in 2010–2014 prior to the modern immune-oncology era [7]. According to the 2019 Fact & Figures publication of the American *Cancer* Society, the 5-year survival rate for non-small cell lung cancer (NSCLC) was 23%, compared to 6% for small-cell lung cancer [8]. Lung cancer survival varies greatly depending on the stage of the disease and the time of diagnosis. According to the seventh edition of the non-small cell lung cancer (NSCLC) TNM groupings, 5-year survival estimates in NSLCC ranged from 73% in stage IA disease to 13% in stage IV disease [9]. A recently published comprehensive literature review investigating changes in lung cancer survival over a four decade-long period confirmed that female patients had a better long-term (5-year) survival rate (17.7%) than male patients (13.0%) [10]. In general, elderly lung cancer patients had poorer survival rates than younger patients. Notably, all age groups have shown a marked increase in survival rates over the decades since the 1970s, particularly in the 15–44 age-group, where 5-year survival exceeded 30% by 2010 [10].

According to the CONCORD-3 study, LC 5-year survival rates fluctuated between 7.7 and 15.7% in East-Central Europe within the 2010–2014 period [7]. Though Hungarian lung cancer incidence is among the highest in Europe [11], to date, there is no data regarding the long-term survival of Hungarian lung cancer patients. Therefore, the objective of our nationwide study was to investigate the 5-year survival rates of patients who were newly diagnosed with LC between 2011 and 2016. Our analysis also aimed to examine the effects of age, gender and the tumors’ histological characteristics on survival as well as to assess the changes in survival rates during the study period.

## Materials and Methods

### Study Design

This nationwide, retrospective study used the claim database of the National Health Insurance Fund of Hungary (NHIF), which is a nationwide insurance system (covering almost 100% of the Hungarian population), as data source. The NHIF database contains medical information regarding ID and ICD-10 codes of the in- and out-patient visits and procedures involved in medical care, containing 100% of lung cancer related intervention as there is no other insurance system for Hungarian citizens for lung cancer treatment. The study was approved by the National Ethical Committee, with the 10338-5/2019/EKU ethical approval number and the I043/88/2019 study license number.

Lung cancer (ICD-10 C34) patients newly diagnosed between January 1, 2011 and December 31, 2016 and ≥20 years or older at the time of diagnosis were included in our study. In order to identify newly diagnosed LC patients from 2011, we set a reference screening period for 2009–2010. Potential miscoding of lung cancer was prevented by only including patients with a minimum of two records of the C34 ICD-10 code within an interval of over 30 but less than 365 days following the first coding. Patients with only one recorded C34 code who died within 60 days after coding, were also included. If patients had ICD-10 codes related to other cancers or if they were administered oncological treatment other than the lung cancer-specific treatment protocol 6 months prior to or 12 months following the first recorded lung cancer code, then these patients were excluded.

Newly diagnosed LC patients were followed up until December 31, 2016 or alternatively until their dates of death, which were also obtained from the NHIF database. Since the immediate cause of death was not available from this data source, all-cause mortality data were accessed. During data collection, data were anonymized and only non-identifiable data were used in the investigation. The total number of newly diagnosed lung cancer patients per year is shown as crude numbers (*n*). For the total number of patients and for both genders separately, the mean ages at diagnosis were determined for the 2011–2012, 2013–2014 and for 2015–2016 diagnostic time intervals. As NHIF database could not provide data on cause specific mortality, therefore we calculated and report overall survival of the Hungarian lung cancer patients as defined by Tan et al. [12]. We investigated survival rates in relation to the pathomorphological types of lung cancer in the patient population, where data were available. Stage of disease was not recorded in the NHIF database. We drew Kaplan-Meier curves to show overall survival. Cox regression model was used estimate the association between gender, age and survival. Interaction between age and gender was also studied. Cox regression was also used to calculate the hazard ratio of death of patients diagnosed between 2015 and 2016 vs. between 2011 and 2012 with adjustment for age. Sex, histology type of lung cancer and main first line treatment type. Interaction between age and period of diagnosis was also studied. All calculations were performed with *R* version 3.5.2 (December 20, 2018) with package boot version 1.3–20.

## Results

### Study Population

As shown in Table 1, 41,860 newly diagnosed lung cancer cases were recorded in the Hungarian NHIF database between 2011 and 2016: 14,080 in 2011–2012, 13,803 in 2013–2014, and 13,977 in 2015–2016. 59.5–62.7% of patients were male, the mean ages of the patients varied between 64.7 and 65.6 years during the study period. The difference between the mean age of male and female LC patients at diagnosis was not clinically relevant.

**TABLE 1:** Patient characteristics.

	2011–2012				2013–2014				2015–2016				Total			
Patients with new LC diagnosis (*n*)	14,080				13,803				13,977				41,860			
Male (*n*, % of LC patients)	8,830		62.71%		8,351		60.50%		8,314		59.48%		**25,495**		**60.91%**	
Female (*n*, % of LC patients)	5,250		37.29%		5,452		39.50%		5,663		40.52%		**16,365**		**39.09%**	
Mean age at diagnosis (y, mean ± SD)	64.88		±10.38		65.28		±10.29		65.76		±9.87		**65.88**		**±9.84**	
Male (y, mean ± SD)	64.67		±9.84		65.11		±9.981		65.69		±9.44		**65.80**		**±9.41**	
Female (y, mean ± SD)	65.22		±11.21		65.55		±10.97		65.86		±10.47		**65.99**		**±10.45**	
Age groups																
20–49	958		6.80%		890		6.45%		724		5.18%		**2,572**		**6.14%**	
50–59	4,070		28.91%		3,766		27.28%		3,396		24.30%		**11,232**		**26.83%**	
60–69	4,966		35.27%		4,985		36.12%		5,617		40.19%		**15,568**		**37.19%**	
70–79	2,983		21.19%		3,035		21.99%		3,165		22.64%		**9,183**		**21.94%**	
80–89	1,027		7.29%		1,038		7.52%		960		6.87%		**3,025**		**7.23%**	
90≤	75		0.53%		89		0.64%		115		0.82%		**279**		**0.67%**	
Morphology																
Squamous cell carcinoma	2,778		19.73%		2,508		18.17%		2,598		18.59%		**7,884**		**18.83%**	
Adenocarcinoma	4,152		29.49%		4,073		29.51%		4,128		29.53%		**12,353**		**29.51%**	
Small cell carcinoma	1,069		7.59%		1,179		8.54%		1,064		7.61%		**3,312**		**7.91%**	
Primary malignancy not specified	6,081		43.19%		6,043		43.78%		6,187		44.27%		**18,311**		**43.74%**	
First line treatment																
Systemic therapy		3,757	26.68%		3,959		28.68%		4,064		29.08%		**11,780**		**28.14%**		
Surgery (including adjuvant therapy)		2,480	17.61%		1,860		13.48%		1,582		11.32%		**5,925**		**14.15%**		

CI, confidence interval; LC, lung cancer; SD, standard deviation. The bold values indicate Patient characteristics.

The highest numbers of patients were recorded in the 60–69 age group (*n* = 15,568; 37.2% of total), peaking at 40.2% by the end of the study period. Patients diagnosed in the 20–49 age group represented 6.1% of the total LC population. The histological type of the tumor was recorded in more than 50% (56.3%) of the cases. The majority of lung cancers was non-squamous cell carcinoma (adenocarcinoma, 29.5%). 7.9% of the total lung cancer population had small-cell carcinoma.

### Long-Term Survival Short and Long-Term Survival

**FIGURE 1:**
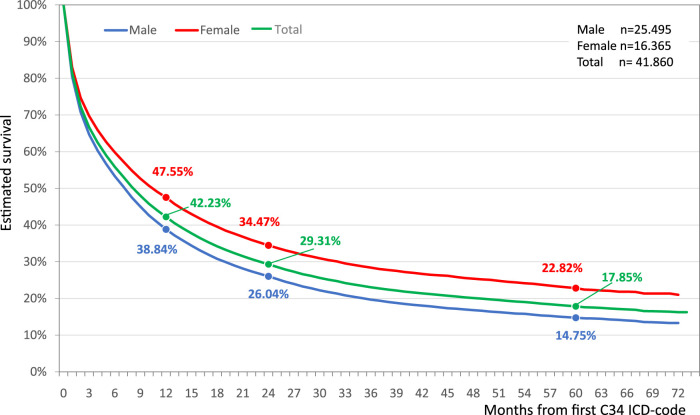
Estimated overall survival of Hungarian lung cancer patients diagnosed between 2011 and 2016.

42.2% of the total lung cancer population (*n* = 41,860) survived the first year. The overall survival rate by the end of the second year the survival rate was 29.3%, and the 5-year survival rate was 17.9% (Figure 1). Female lung cancer patients had better survival throughout the study period. Female patients had a 47.6% one-year survival rate vs. 38.8% for males. 5-year survival rates for females and males were 22.8 and 14.8%, respectively (Figure 1).

**FIGURE 2:**
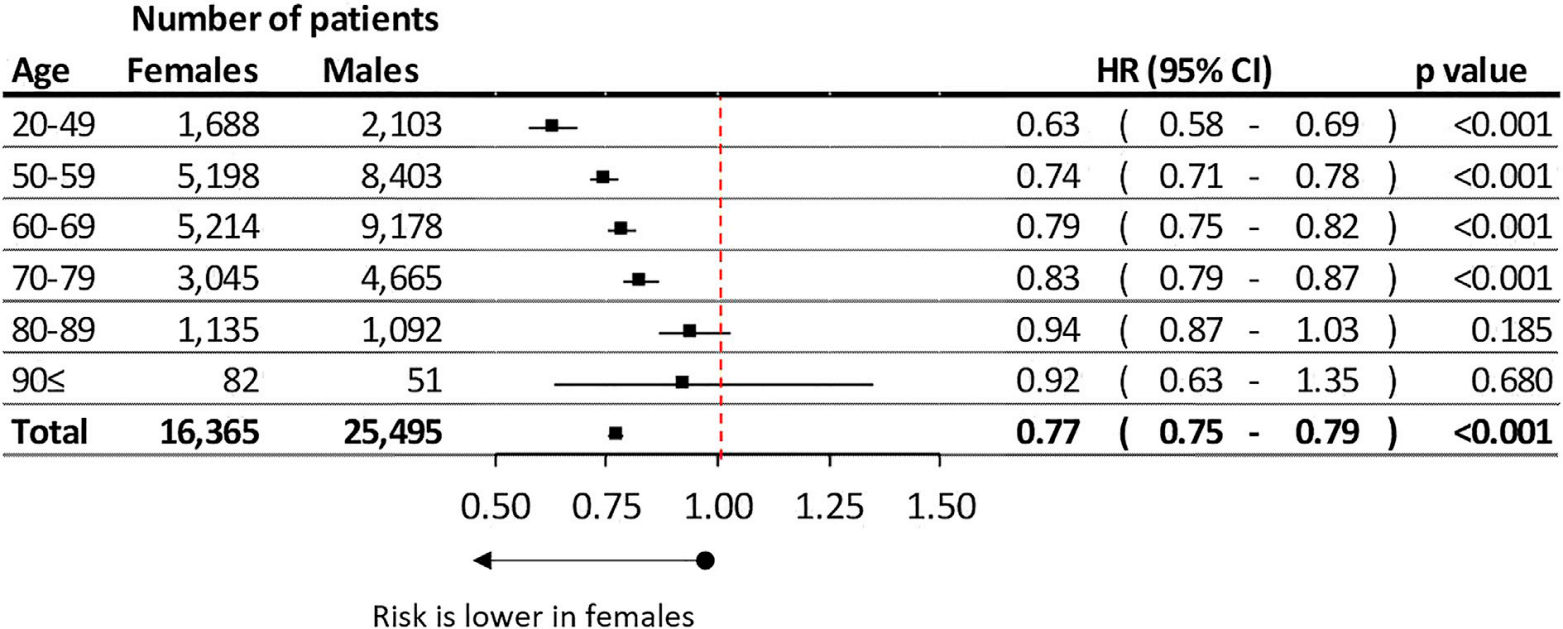
Difference of estimated overall lung cancer survival between female and male in different age groups.

Females had 22.88% better lung cancer survival compare to males (HR: 0.77; 95% CI: 0.75–0.79, *p* < 0.001) (Figure 2). The difference in survival between females and males increased in the younger age groups reaching 37.16% in the youngest cohort. While these differences decreased to 21% and 17% in the 60–69 and 70–79 age groups. The survival differences between the genders were found to be non-significant above the age of 80 (Figure 2).

One-year survival rates were highest in the 20–49 age group, reaching 55.1%, while the largest 50–59 and 60–69 age cohorts had 48.3 and 44.1% survival rates by the end of the first follow-up year. The long-term survival rates decreased to 31.3, 22.5, 17.7, 10.2% in the 20–49, 50–59, 60–69 and 70–79 age cohorts, respectively (Supplementary Figure S1).

**FIGURE 3:**
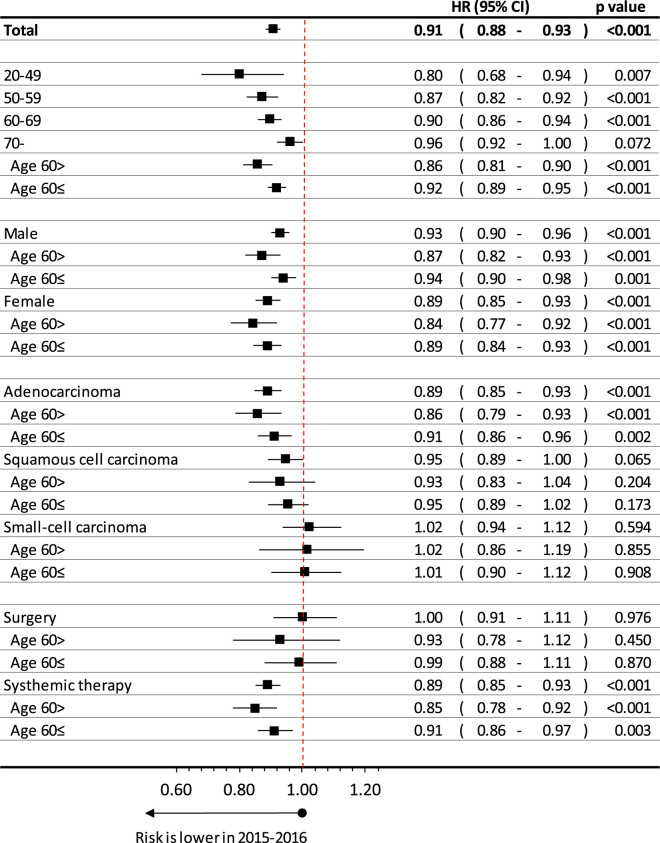
Age, sex, histology type and treatment dependent difference in estimated overall lung cancer survival between patients diagnosed in 2015–2016 and in 2011–2012.

Lung cancer patients diagnosed between 2015 and 2016 had a 5.3% lower risk of mortality compared to patients in the 2011–2012 study period (HR 0.95 95% CI 0.92–0.97; *p* = 0.001) adjusted only for age (Figure 3). When we adjusted the change of survivals by age, sex, histology type of lung cancer and main treatment type, we got 9.2% improvement (HR = 0.91; 95%CI: 0.88–0.93; *p* < 0.001).

The improvement of survival was higher by younger age, where *p* for trend analysis was significant, interaction *p* = 0.014. We also calculated this interaction between age above or below 60 years: interaction: *p* = 0.024 interaction (period and type): *p* = 0.003 (These results were adjusted for sex, histology type of lung cancer and treatment types).

Improvement of survival was found to be higher at females (HR:0.89; 95%CI:0.85–0.93; *p* < 0.001) than males (HR: 0.93; 95%CI:0.90–0.96; *p* < 0.001), especially at younger age, thought the interaction analysis for sex was not significant (*p* = 0.1024) (These results were adjusted for age (expect detailed age perspective) histology type of lung cancer and treatment types).

When we compared the 2015–16 and 2011–2012 period by histology type of lung cancer, we found the highest improvement at adenocarcinoma, where hazard ratio was 0.89 (95%CI: 0.85–0.93; *p* < 0.001), especially under age of 60 years, where HR was 0.86 (95%CI:0.79–0.93; *p* < 0.001). On the other hand, we recorded 5%, but not significant improvement at patients having squamous cell carcinoma (HR:0.95; 95%CI:0.89–1.00; *p* = 0.065), while there were no change found at small-cell carcinoma (HR: 1.02; 95%CI: 0.94–1.12; *p* = 0.594). Interaction analysis showed significant relation for type of histology (*p* = 0.003) (These results were adjusted for sex, age (expect age related parts) of lung cancer patients and treatment types).

We also analyzed those lung cancer patients having systemic treatment in late stage or surgery in early stage. At those, whom had surgery in first line treatment, improvement were not detected (HR:1.00; 95%CI: 0.91–1.11; *p* = 0.976), while we recorded relevant increase in survival at patients received systemic therapy (HR:0.89; 95%CI:0.85–0.93; *p* < 0.001), especially those, were age below 60 years (HR:0.85; 95%CI:0.78–0.92); *p* < 0.001).

**FIGURE 4:**
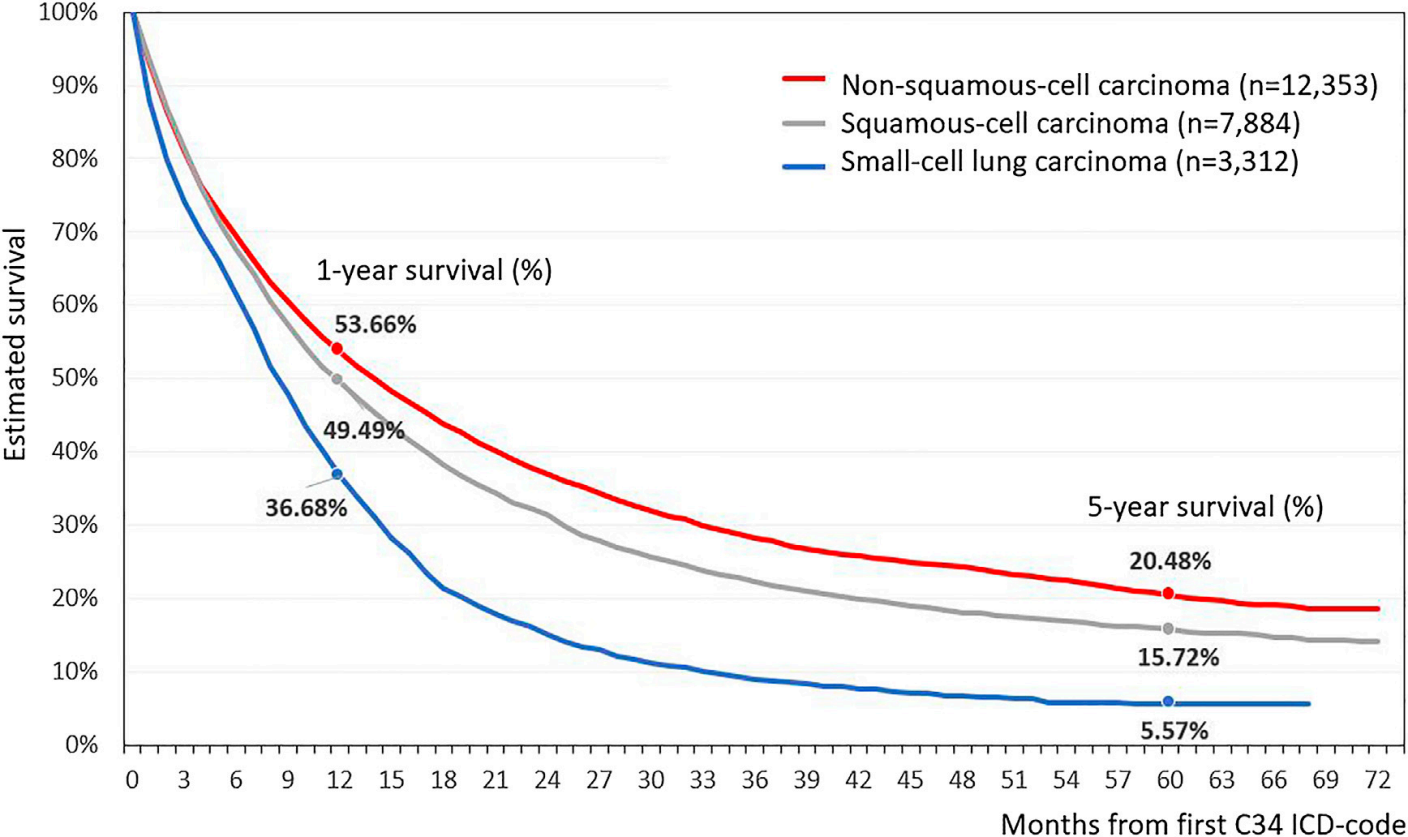
Estimated survival of Hungarian lung cancer patients depending on histological characteristics.

Based on the morphological report of the NHIF, the histological type of lung cancer was recorded in 56.3% of all LC patients. Adenocarcinoma was more common in females, with 33.4% vs. 27.0% in male patients, while squamous-cell carcinoma was more frequently found in the male patient population, 22.7% in males vs. 12.9% in females (Supplementary Table S1). The best crude survival rates among LC patients could be found in patients with adenocarcinoma (*n* = 12,353), where the 1- and 5-year survival rates were 53.7 and 20.5%, respectively (Figure 4). The poorest survival rates were recorded in the small-cell lung carcinoma group (*n* = 3,312) where the 5-year survival proved to be only 5.6%.

We could record the 1^st^ line treatment as systemic therapy at 28.14% of all patients (including chemo and targeted therapy), and surgery (including those having adjuvant treatment) at 14.15%. The share of systemic therapy increased from 26.68 onto 29.08%, while share of those having surgery in early stage, decreased from 17.60 onto 11.32%.

## Discussion

Our retrospective, longitudinal analysis provides long-term LC overall survival data from Hungary based on a comprehensive, nationwide data source. This study is also the first to report an improvement in LC survival during the 2011–2016 period in Hungary. The main findings of this large-scale evaluation can be summarized as follows:

1. Hungarian LC patients had a 5-year overall survival rate of 17.9% between 2011 and 2016, with a 5.3% improvement in survival during the 6 year-study period.
2. The survival of LC depends on age and sex, as well as on cancer type. Females had a 23% lower mortality risk than male LC patients, and the highest crude survival rates were found in patients with adenocarcinoma.

### Short-Term Survival of Lung Cancer

Despite several recently published analyses on lung cancer in Europe [10–13], neither short-term (1-year) nor long-term (5Y) data have been available regarding survival in Hungarian patients. When we compared our short-term survival results with findings from the Danish Lung *Cancer* Group study [14], one-year survival rates were comparable (42.37% vs. 43% for the Hungarian 2011–2012 and Danish 2010–2012 interval, respectively), although the Danish study used a different diagnostic period. Additionally, the drop of the survival curve of Hungarian LC patients was more rapid and occurred earlier. The distribution of male and female 1-year survival rates was also similar, 39.2% vs. 39% in Hungarian and Danish males, and 47.66% vs. 47% in Hungarian and Danish females, respectively. The short-term survival rates in our study were unequivocally higher than the EUROCARE-5 study reported in Eastern European countries (32.4%) [15]. They corresponded with the Central European country results (42.3%), although this study analyzed patients diagnosed between 2000 and 2007 [15]. The *Cancer* Research United Kingdom database provided short-term LC survival data for both genders in the 2010–2011 diagnostic period [16], where net survival rates were 30.4% in males and 35.1% in females, nevertheless, LC survival rates in the United Kingdom tended to be at the lower end of rates collected in Western European countries [7–15].

### Long-Term Survival of Lung Cancer

Studies involving patients diagnosed with lung cancer before 2014 were the only data source which could provide 5-year survival data in 2019. Hence, a number of international publications and data sources were of limited comparability with regard to our study encompassing the 6 year period of 2010–2016. In the CONCORD-3 study, the age-standardized 5-year survival rates varied between 7.7 and 15.7% for East-Central Europe in the 2010–2014 diagnostic period [7]. In our study, we observed a 17.9% 5-year survival rate in the 2010–2011 diagnostic period. Since our defined age cohorts were slightly different than what the CONCORD-3 study used for standardization, we were not able to directly compare our national results with other East-Central European countries. Nevertheless, with a similar age distribution in our study population as well as in neighboring countries, Hungarian survival rates could be considered comparable with the survival rates of countries in the region and in most Western European countries. These similar numbers could be due to the same, limited LC treatment options of the early 2010s as well as the advanced stage of LC at diagnosis. Gender-specific survival data for comparison were only available from the *Cancer* Research United Kingdom database [16] where the 5-year net survival rates were 8.4 and 11.6% in males and females, respectively. These results were lower compared to our findings (14.75 and 22.84% in males and in females), although the gender differences tended to be similar between the two analyses.

### Trends in Long Term Survival

We demonstrated a 5.3% improvement in survival (9.0% adjusted) between 2015–2016 and 2010–2011 periods, which was an increase expressed by hazard ratio, hence not the change of the fifth year survival rates as 2015–2016 period did not have such a long follow-up period. Nevertheless, the change is close to a yearly 1% improvement. If we compare the trends of lung cancer survival in other study, we can find a 18.9% vs 12.4% in Denmark, 14.7% vs 10.0% in United Kingdom, 20.4% vs 15.4% in Norway and 21.7% vs 18.5% in Canada by comparing the 2010–2014 and 2005–2009 periods based on the ICBP SURVMARK-2 study [17]. In this aspect, this is a 3–6% absolute change of fifth year net survival within a 10-year period, which is hardly comparable with the trend of our survival improvement in a 6-year period, though these changes are in the same range. The US SEER database presented the fifth year survival change from 18.42 onto 21.41% from 2007 to 2012, in a same, 6-year period, which is an average 2.1% yearly improvement [18]. If we investigate the trends of fifth year lung cancer survival in the CONCORD-3 study, we can see a 0.8% absolute change in Czech, 0.7% in Slovakia and 0.3% in Poland between 2010–2014 and 2005–2009 period, which is a 8.16, 6.67, and 2.13% relative change consequently where the Slovak and Czech results are comparable with our improvement [7]. Nevertheless, in the Western EU countries (like Denmark, Norway, United Kingdom, Ireland), CONCORD-3 study presented even a 25–35% relative improvement in the relative change of 5^th^ year survival during this period. All in all, we can conclude that the size of lung cancer survival improvement is within the range of previously presented study results, even if the data are hardly comparable. Besides, if we expect the at least the same improvement rate of the Hungarian lung cancer survival in last years, the 5^th^ year survival may exceed the 20% by the end of this decade, especially due to the impact of newly approved immunotherapies.

### Gender Differences in Lung Cancer Survival

We found 23% better survival rates for female LC patients than for males, and survival differences tended to be larger (up to 37%) in the younger age groups. Gender differences have been investigated in several studies during the past decades, like in the analysis from the US lung cancer database [8]. Average survival rates were 13.0% in males and 17.7% in females. The ECOG 1594 trial involving 1,157 patients found a statistically significant, 1.9-month improvement in median survival among women compared to men, despite similar response rates and greater toxicity from treatment and no differences regarding other known prognostic factors [19]. Population-based studies have supported this finding, including a multivariate analysis of more than 20,000 lung cancer cases from Poland that found a 1.15 relative risk (RR) of death in men [20]. An evaluation of 4,618 NSCLC patients who were prospectively enrolled and followed from 1997 to 2002 at the Mayo Clinic in Rochester, Minnesota found a 1.20 relative risk of mortality in men after adjusting for age, histology, cancer stage, smoking history, and treatment [21]. These differences in survival between genders may be multifactorial [22]. Due to gender differences in smoking habits, females were prone to develop adenocarcinoma more frequently [ [23–25]] which LC lung type results in better survival rates than other histological types of LC [ [26, 27]]. Our analysis confirmed that adenocarcinoma was more frequently diagnosed in females than in men. Additionally, females tended to have higher rates of surgical treatment in the early stages of lung cancer [28], which resulted in improved survival outcomes in these stages compared to other modalities of treatment [29].

On the other hand, though the improvement in survival was higher at females, especially at younger age, the interaction analysis was not significant for sex, therefore we could conclude that sex did not play role in the time dependent improvement of lung cancer.

### Age-Dependent Survival of Lung Cancer

Older lung cancer patients had poorer survival than younger patients [8]. All age groups have shown significant improvements in survival rates since the 1970s, and the greatest improvements were recorded in the youngest age groups (15–44 years), as reported by Lu et al. [10]. Our findings have confirmed these trends, since the youngest lung cancer patients had 31.3% crude 5-years survival rate, compared to 10.2% in the age group of 70–79 years. We found more pronounced improvements in age-dependent survival rates at younger cohorts during the 2015–2016 interval than in the 2011–2012 diagnostic period. Though we could not identify the prevalence of typical carcinoids at young cohort, it may also play role in better survival results of this cohort due to its good prognosis and curability [30].

### Long-Term Survival of Lung Cancer by Cancer Type

Lung cancer survival is known to vary depending on the histological type of the cancer. Patients with adenocarcinoma have been shown to have higher survival rates compared to those with squamous cell carcinoma, while patients with small-cell carcinoma have been found to have the lowest 5-year survival rates compared to the previous two LC subtypes [10, 31]. In our study, the histological types of lung cancer could be identified in 56.3% of the cases. 5-year survival rates among patients with adenocarcinoma and those with squamous cell carcinoma were 20.5 and 15.7%, respectively, and were comparable with international findings [10]. In line with previous observations, adenocarcinoma occurred more frequently while squamous cell carcinoma less frequently in females.

We found the highest improvement of survival in case of adenocarcinoma (11%), especially those, having age below 60 years at time of diagnosis. On the other hand, we did not find any change in case of small cell carcinoma, while relevant, 5%, but not significant at squamous cell carcinoma. This result is parallel with the historic changes in the treatment of different types of lung cancer. The aera of personalized treatment, the introduction of targeted therapy was most frequent at adenocarcinoma, especially at young patients (ALK inhibitors), while in older women, the possibility of EGFR inhibition resulted in visible improvement [32]. On the other hand, we did not have any significant change in treatment options patients with small cell carcinoma between 2011 and 2016. Nevertheless, these types of changes reflected in a 11% overall change in survival of patient population received systemic therapy, while we could not find relevant improvement at those, having surgery in first line treatment.

Strength of our study is the large number of diagnosed lung cancer patients, the carefully cleaned data, the 6-year-long follow-up period and the nationwide nature of the NHIF database, which all provide a solid foundation for drawing conclusions from our analysis. However, our study is not free from limitations. The applied exclusion criteria may have led to the exclusion of patients who had other cancer types besides lung cancer. According to our estimations however, this patient population is negligible. Moreover, the NHIF database contained data on the pathomorphology of lung cancer in only 52% of the cases. Staging, ECOG status and laboratory tests of patients were not recorded in the NHIF database. Consequently, we were not able to provide specific survival data based on these characteristics. Other limitation of our study is that we were able to provide only overall survival of lung cancer patients, and we did not estimate net survival, hence, we could not adjust the age related survival analysis to the natural survival rates of the general population. Nevertheless, as lung cancer patients have much higher mortality than the reference general population, and the vast majority of their mortality is caused by the cancer itself, this does not have a severe effect on the results and interpretation. Beside, we were able to detect the type of first treatment only certain part of lung cancer population. Those, having first line treatment as part of a study or received radiotherapy or palliative care, were not recorded in the NHIF database, hence we evaluated those, whom 1^st^ line treatment was unambiguous, like systemic therapy or surgery and only from the perspective of survival improvement analysis.

## Conclusion

To summarize, our study is the first to provide long-term survival data on Hungarian lung cancer patients and to report a 9.2% adjusted improvement in survival during the 6-year study period. The 5-year crude survival rate of 17.8% is comparable with neighboring countries’ data from the same period. Female lung cancer patients had a 23% better survival rate than males, which could be attributed to a higher incidence rate of adenocarcinoma in women. Survival rates were comparable to–and at the higher end of–rates registered in other East-Central European countries.

## Data Availability

The raw data supporting the conclusions of this article will be made available by the authors, without undue reservation.

## References

[1] Ding L Getz G Wheeler DA Mardis ER McLellan MD Cibulskis K Somatic mutations affect key pathways in lung adenocarcinoma. Nature (2008). 455:1069–75. 10.1038/nature07423 18948947 PMC2694412

[2] Ferlay J Colombet M Soerjomataram I Dyba T Randi G Bettio M Cancer incidence and mortality patterns in Europe: estimates for 40 countries and 25 major cancers in 2018. Eur J Cancer (2018). 103:356–87. 10.1016/j.ejca.2018.07.005 30100160

[3] Hakulinen T Engholm G Gislum M Storm HH Klint Å Tryggvadóttir L Trends in the survival of patients diagnosed with cancers in the respiratory system in the Nordic countries 1964-2003 followed up to the end of 2006. Acta Oncologica (2010). 49(5):608–23. 10.3109/02841860903575281 20170292

[4] Torre LA Siegel RL Jemal A Lung cancer statistics. Adv Exp Med Biol (2016). 893:1–19. 10.1007/978-3-319-24223-1_1 26667336

[5] Jemal A Siegel R Xu J Ward E Cancer statistics, 2010. CA Cancer J Clin (2010). 60(5):277–300. 10.3322/caac.20073 20610543

[6] Heuvers ME Hegmans JP Stricker BH Aerts JG Improving lung cancer survival; time to move on. BMC Pulm Med (2012). 12:77. 10.1186/1471-2466-12-77 23234250 PMC3528634

[7] Allemani C Matsuda T Di Carlo V Harewood R Matz M Nikšić M Global surveillance of trends in cancer survival 2000-14 (CONCORD-3): analysis of individual records for 37 513 025 patients diagnosed with one of 18 cancers from 322 population-based registries in 71 countries. Lancet (2018). 391(10125):1023–75. 10.1016/S0140-6736(17)33326-3 29395269 PMC5879496

[8] American Cancer Society. Cancer facts & figures 2018. Atlanta: American Cancer Society (2018).

[9] Woodard GA Jones KD Jablons DM Lung cancer staging and prognosis. Cancer Treat Res (2016). 170:47–75. 10.1007/978-3-319-40389-2_3 27535389

[10] Lu T Yang X Huang Y Zhao M Li M Ma K Trends in the incidence, treatment, and survival of patients with lung cancer in the last four decades. Cmar (2019). 11:943–53. 10.2147/cmar.s187317 PMC6345192 30718965

[11] Bogos K Kiss Z Gálffy G Tamási L Ostoros G Müller V Revising incidence and mortality of lung cancer in central Europe: an epidemiology review from Hungary. Front Oncol (2019). 9:1051. 10.3389/fonc.2019.01051 31709174 PMC6819432

[12] Tan KS Eguchi T Adusumilli PS Reporting net survival in populations: a sensitivity analysis in lung cancer demonstrates the differential implications of reporting relative survival and cause-specific survival. Clep (2019). 11:781–92. eCollection 2019. 10.2147/CLEP.S210894 PMC6730547 31564983

[13] Arnold M Rutherford MJ Bardot A Ferlay J Andersson TM Myklebust TA Progress in cancer survival, mortality, and incidence in seven high-income countries 1995-2014 (ICBP SURVMARK-2): a population-based study. Lancet Oncol (2019). 20(11):1493–505. 10.1016/S1470-2045(19)30456-5 31521509 PMC6838671

[14] Jakobsen E Rasmussen TR Green A Mortality and survival of lung cancer in Denmark: results from the Danish lung cancer group 2000-2012. Acta Oncologica (2016). 55(Suppl. 2):2–9. 10.3109/0284186x.2016.1150608 27056247

[15] Rossi S Baili P Capocaccia R Caldora M Carrani E Minicozzi P The EUROCARE-5 study on cancer survival in Europe 1999-2007: database, quality checks and statistical analysis methods. Eur J Cancer (2015). 51(15):2104–19. 10.1016/j.ejca.2015.08.001 26421815

[16] Cancer Research UK (2019). https://www.cancerresearchuk.org/health-professional/cancer-statistics/statistics-by-cancer-type/lung-cancer/survival#heading-Zero (Accessed January 24, 2020).

[17] Arnold M Rutherford MJ Bardot A Ferlay J Andersson TM-L Myklebust TÅ Progress in cancer survival, mortality, and incidence in seven high-income countries 1995-2014 (ICBP SURVMARK-2): a population-based study. Lancet Oncol (2019). 20(11):1493–505. 10.1016/s1470-2045(19)30456-5 31521509 PMC6838671

[18] National Cancer Institute. Surveillance, epidemiology and end result program. Bethesda: (SEER database): cancer stat facts: lung and bronchus cancer (2020). https://seer.cancer.gov/statfacts/html/lungb.html. Last update (Accessed October 31, 2020).

[19] Wakelee HA Wang W Schiller JH Langer CJ Sandler AB Belani CP Survival differences by sex for patients with advanced non-small cell lung cancer on eastern cooperative oncology group trial 1594. J Thorac Oncol (2006). 1(5):441–6. 10.1016/s1556-0864(15)31609-9 17409897

[20] Radzikowska E Głaz P Roszkowski K Lung cancer in women: age, smoking, histology, performance status, stage, initial treatment and survival. Population-based study of 20 561 cases. Ann Oncol (2002). 13:1087–93. 10.1093/annonc/mdf187 12176788

[21] Visbal AL Williams BA Nichols FC Marks RS Jett JR Aubry M-C Gender differences in non-small-cell lung cancer survival: an analysis of 4,618 patients diagnosed between 1997 and 2002. Ann Thorac Surg (2004). 78:209–15. 10.1016/j.athoracsur.2003.11.021 15223430

[22] Rivera M Lung cancer in women: differences in epidemiology, biology, histology, and treatment outcomes. Semin Respir Crit Care Med (2013). 34(6):792–801. 10.1055/s-0033-1358550 24258569

[23] Devesa SS Bray F Vizcaino AP Parkin DM International lung cancer trends by histologic type: male:female differences diminishing and adenocarcinoma rates rising. Int J Cancer (2005). 117(2):294–9. 10.1002/ijc.21183 15900604

[24] Meza R Meernik C Jeon J Cote ML Lung cancer incidence trends by gender, race and histology in the United States, 1973-2010. PLoS One (2015). 10(3):e0121323. 10.1371/journal.pone.0121323 25822850 PMC4379166

[25] Welcker K Genderspezifische Unterschiede des Lungenkarzinoms. Zentralbl Chir (2015). 140(3):260–5. 10.1055/s-0034-1396231 25906024

[26] Ramalingam S Dinan MA Crawford J Survival comparison in patients with stage IV lung cancer in academic versus community centers in the United States. J Thorac Oncol (2018). 13(12):1842–50. 10.1016/j.jtho.2018.09.007 30312680

[27] Kinoshita FL Ito Y Morishima T Miyashiro I Nakayama T Sex differences in lung cancer survival: long-term trends using population-based cancer registry data in Osaka, Japan. Jpn J Clin Oncol (2017). 47(9):863–9. 10.1093/jjco/hyx094 28903532

[28] Fu JB Kau TY Severson RK Kalemkerian GP Lung cancer in women. Chest (2005). 127(3):768–77. 10.1378/chest.127.3.768 15764756

[29] Cerfolio RJ Bryant AS Scott E Sharma M Robert F Spencer SA Women with pathologic stage I, II, and III non-small cell lung cancer have better survival than men. Chest (2006). 130(6):1796–802. 10.1378/chest.130.6.1796 17166999

[30] Velinovic M Jankovic R Jovanovic D Skodric Trifunovic V Gavrilovic D Stojsic J Tumor characteristics, expressions of ERCC1, Bax, p53, IGF1R, Bcl2, Bcl2/Bax and prognostic factors for overall survival in patients with lung carcinoid. J BUON (2019). 24(1):256–66. 30941978

[31] Walters S Maringe C Coleman MP Peake MD Butler J Young N Lung cancer survival and stage at diagnosis in Australia, Canada, Denmark, Norway, Sweden and the UK: a population-based study, 2004-2007. Thorax (2013). 68(6):551–64. 10.1136/thoraxjnl-2012-202297 23399908

[32] Duma NN Santana-Davila JR Molina JR Non-small cell lung cancer: epidemiology, screening, diagnosis, and treatment. Mayo Clinic Proc (2019). 94(8):1623–40. 10.1016/j.mayocp.2019.01.013 31378236

