# Supplementary material for: Improvement in Lung Cancer Survival: 6-Year Trends of Overall Survival at Hungarian Patients Diagnosed in 2011–2016
Source: Pathol Oncol Res. 2021 Apr 30;27:603937. doi: 10.3389/pore.2021.603937 (PMC8262181; doi:10.3389/pore.2021.603937)
Supplement: Supplementary file 1 [file Table1.docx]

|  | **Male** | | **Female** | | **Total** | |
| --- | --- | --- | --- | --- | --- | --- |
|  | **number** | **% of total** | **number** | **% of total** | **number** | **% of total** |
| Total | 25,495 | 100.00% | 16,365 | 100.00% | **41,860** | **100.00%** |
| Adenocarcinoma | 6,882 | 26.99% | 5,471 | 33.43% | **12,353** | **29.51%** |
| Squamous carcinoma | 5,774 | 22.65% | 2,110 | 12.89% | **7,884** | **18.83%** |
| Small-cell carcinoma | 1,888 | 7.41% | 1,424 | 8.70% | **3,312** | **7.91%** |
| Not specified during histology | 3,997 | 15.68% | 2,641 | 16.14% | **6,638** | **15.86%** |
| Not recorded | 6,953 | 27.27% | 4,719 | 28.84% | **11,672** | **27.88%** |
