# Supplementary figures and images for: Improvement in Lung Cancer Survival: 6-Year Trends of Overall Survival at Hungarian Patients Diagnosed in 2011–2016
Source: Pathol Oncol Res. 2021 Apr 30;27:603937. doi: 10.3389/pore.2021.603937 (PMC8262181; doi:10.3389/pore.2021.603937)

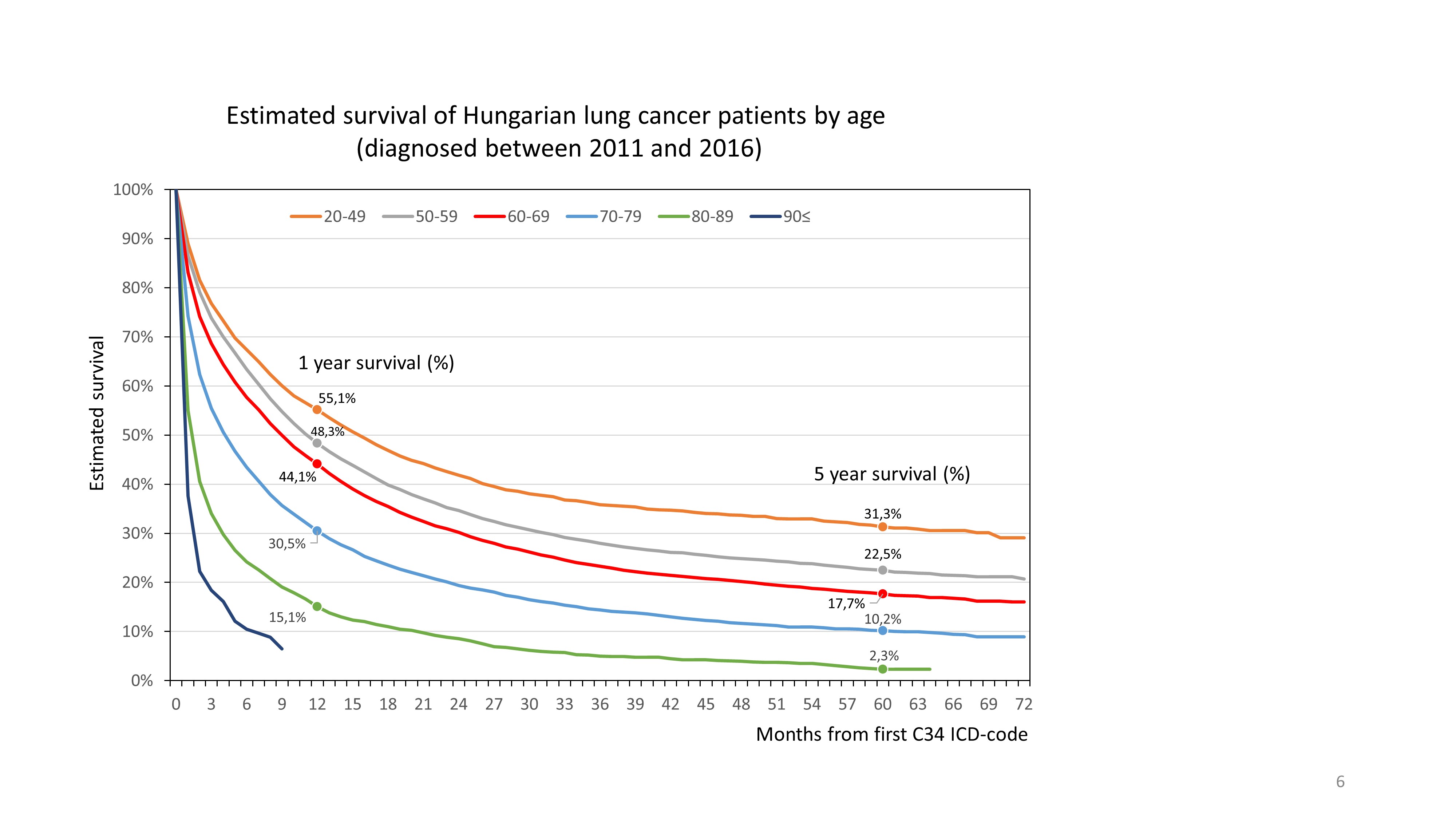

Supplement: Supplementary file 2 [file Image1.jpg]
